# The effects of high‐intensity exercise training and detraining with and without active recovery on postexercise hypotension in young men

**DOI:** 10.14814/phy2.15862

**Published:** 2023-12-21

**Authors:** Tze‐Huan Lei, Naoto Fujii, Xiao Zhang, Faming Wang, Toby Mündel, I‐Lin Wang, Yi‐Ming Chen, Takeshi Nishiyasu, Tatsuro Amano, Kohei Dobashi, Lin Wang, Tzu‐Shao Yeh, Narihiko Kondo, Richie P. Goulding

**Affiliations:** ^1^ College of Physical Education Hubei Normal University Huangshi China; ^2^ Institute of Health and Sport Sciences University of Tsukuba Tsukuba Japan; ^3^ Shanghai Normal University Kangcheng Experimental School Shanghai China; ^4^ Division Animal and Human Health Engineering, Department of Biosystems (BIOSYST) KU Leuven Leuven Belgium; ^5^ Department of Kinesiology Brock University St. Catharines Ontario Canada; ^6^ Department of Food Science Fu Jen Catholic University New Taipei City Taiwan; ^7^ Faculty of Education Niigata University Niigata Japan; ^8^ Faculty of Education Hokkaido University of Education Asahikawa Japan; ^9^ School of Physical Education Wuhan University of Technology Wuhan China; ^10^ School of Public Health Nantong University Nantong China; ^11^ Laboratory for Applied Human Physiology, Graduate School of Human Development and Environment Kobe University Kobe Japan; ^12^ Laboratory for Myology, Department of Human Movement Sciences, Faculty of Behavioral and Human Movement Sciences Vrije Universiteit, Amsterdam Movement Sciences Amsterdam the Netherlands

**Keywords:** arterial baroreflex, physical training and detraining, postexercise hypotension

## Abstract

Whether high‐intensity exercise training and detraining combined with skeletal muscle pump (MP) could alter the magnitude of postexercise hypotension has not been investigated. We therefore sought to determine whether the combination of MP (unloaded back‐pedaling) with 4 weeks of high‐intensity exercise training and detraining could alter the magnitude of postexercise hypotension. Fourteen healthy men underwent 4 weeks of high‐intensity exercise training (5 consecutive days per week for 15 min per session at 40% of the difference between the gas exchange threshold and maximal oxygen uptake [i.e., Δ40%]) followed by detraining for 4 weeks. Assessments were conducted at Pre‐training (Pre), Post‐training (Post) and after Detraining with (MP) and without MP (Con). The exercise test in the Pre, Post and the Detraining consisted of 15 min exercise at Δ40% followed by 1 h of recovery. At all time‐points, the postexercise reduction in mean arterial pressure (MAP) was reduced in MP compared to Con (all *p* < 0.01). Four weeks of high‐intensity exercise training resulted in a reduction in the magnitude of postexercise hypotension (i.e., the change in MAP from baseline was mitigated) across both trials (All *p* < 0.01) when compared to Pre and Detraining. Following Detraining, the reduction of MAP from baseline was reduced compared to Pre, but was not different from Post. We conclude that high‐intensity exercise training combined with skeletal MP reduces the magnitude of postexercise hypotension, and this effect is partially retained for 4 weeks following the complete cessation of high‐intensity exercise training.

## 1 INTRODUCTION

Following dynamic endurance‐type exercise, a reduction in mean arterial blood pressure (MAP) occurs relative to resting values (i.e., postexercise hypotension), lasting from 1 to 24 h (Graham et al., 2016; Pescatello et al., 1991). Postexercise hypotension occurs due to the combined effects of postexercise skeletal muscle vasodilation and a centrally‐mediated reduction in sympathetic outflow from the rostral ventrolateral medulla to the sympathetic adrenergic nerves, leading to a reduction in vasoconstrictor activity (Chen & Bonham, 2010).

Postexercise hypotension can result in postexercise syncope in healthy individuals if occurring without maintenance of the muscle pump (MP, and hence, venous return) when stood in an upright position. Roughly 50%–80% of healthy individuals develop pre‐syncopal symptoms if standing motionless following dynamic exercise (Halliwill et al., 2014). Regular aerobic training and active recovery following dynamic exercise have been shown to prevent the drop in arterial blood pressure during an orthostatic challenge (Winker et al., 2005) and when compared with no active recovery (Carter 3rd et al., 1999).

To date, the studies with postexercise hypotension mainly focus on the acute effect of postexercise hypotension (MacDonald et al., 1999; Meade et al., 2018; Mündel et al., 2015), whereas whether the magnitude of postexercise hypotension can be altered by different training statuses using within‐subject design remains unknown. In fact, aerobic training status has long been suggested to influence the magnitude of postexercise hypotension; however, experimental data confirming this hypothesis are scant and vary considerably from each other. For example, Bonsu and Terblanche (2016) showed that the magnitude of postexercise hypotension was greater in obese women following six sessions of high‐intensity exercise training, whereas Rivas et al. (2019) showed that 10 days of training in a temperate environment resulted in a lower magnitude of postexercise hypotension during the final 30 min of recovery. Such inconsistent findings might be due to the fact that Bonsu and Terblanche (2016) included an obese population, whilst Rivas et al. (2019) studied a healthy population. It is well known that muscle sympathetic and baroreflex sensitivity is greater in lean than obese subjects (Grassi et al., 1998), and so the greater magnitude of postexercise hypotension following training could be due to differences in body composition *per se* and not due to training status. In fact, these two studies did not quantify the effect on training status, as they did not measure *V̇*O_2_ max before and after physical training. Due to this reason, the effect of altered training status on postexercise hypotension remains to be explored.

A potential physiological mechanism for increasing the baroreflex during the recovery following exercise training documented by Rivas et al. (2019) could be a reduction in the postexercise hyperthermic response, which subsequently resulted in a reduced postexercise hyperaemic response (Elsner & Carlson, 1962). A lower postexercise hyperaemic response, combined with a lower core temperature during the recovery period, could subsequently reduce the magnitude of postexercise vasodilation of the skeletal vasculature (Elsner & Carlson, 1962; Kapilevich et al., 2020; Pearson et al., 2011), and thereby facilitate greater venous return. However, this supposition has not yet been tested.

Although Rivas et al. (2019) demonstrated that 10 days of training in a thermoneutral environment was able to lower the magnitude of postexercise hypotension during recovery, this study did not provide any insight into the cardiovascular adaptations that may have underpinned these findings. Moreover, an additional means of testing the influence of physiological adaptations associated with training status on postexercise hypotension would be to measure subjects again following the cessation of training (i.e., detraining); however, this was not performed. Therefore, the independent effect of physiological adaptations associated with an elevated training status cannot be determined. As high‐intensity exercise training has been demonstrated to maintain sympathovagal balance following 60‐day of bed rest (Maggioni et al., 2018), this interventional strategy could potentially be used to examine the effect of alterations in training status on postexercise hypotension using a within‐subject design.

The effect of detraining on postexercise hypotension has not been investigated in healthy participants to date. It was previously demonstrated that short‐duration, high‐intensity exercise training performed during a 60‐day period of bed rest enabled the maintenance of cardiac sympathovagal balance during orthostatic stress (Maggioni et al., 2018). Hence, this raises the possibility that improvements in cardiac sympathovagal balance (and hence, any effects of training on the postexercise hypotension response) resulting from short‐term high‐intensity exercise training may be maintained following an equivalent period of detraining. Given the globally rising levels of physical inactivity and recent COVID‐19 pandemic, whether high‐intensity training can mitigate changes in the magnitude of postexercise hypotension induced by a period of deconditioning remains a societally important issue.

Maintenance of the skeletal MP following dynamic exercise reduces the magnitude of postexercise hypotension by reducing venous pressure and blood volume of the exercising limb, thereby increasing venous return and offsetting reductions in MAP following dynamic exercise (Carter 3rd et al., 1999). To date, studies have mainly focused on the acute effects of skeletal MP on postexercise hypotension in either temperate (Carter 3rd et al., 1999) or hot environments (Gagnon et al., 2008), and thus the effects of the MP with those of physical training and detraining on postexercise hypotension remain completely unexplored. Furthermore, whether high‐intensity exercise training combined with MP can completely nullify the occurrence of postexercise hypotension has not been investigated to date. By examining the effect of MP when combined with different training statuses, this will further test the hypothesis that postexercise hypotension is affected by different training statuses, as if high‐intensity training is believed to reduce the magnitude of postexercise hypotension, the increase of baroreflex should be greater in the trial with than without the effect of MP.

The aims of this study were as follows: (1) to determine the effects of a 4‐week high‐intensity exercise training intervention on the magnitude of postexercise hypotension; (2) to determine whether any changes in the magnitude of postexercise hypotension brought about via training would be preserved following a 4‐week detraining period; and (3) to determine the influence of the MP on postexercise hypotension following training and detraining. We hypothesized that: (1) the magnitude of postexercise hypotension would be lowered following 4‐week training; and (2) that the magnitude of postexercise hypotension would be retained following 4‐week detraining with respect to baseline. As the skeletal MP has been shown to reduce the magnitude of postexercise hypotension, we further hypothesized that (3) 4‐week of training combined with skeletal MP would not result in any postexercise hypotensive response throughout the recovery.

## 2 METHODS

### 2.1 Participants

An a priori power analysis (G*Power version 3.1.9.4; Heinrich Heine University Düsseldorf, Düsseldorf, Germany) showed that a minimum of 8 participants was required based on conventional *α* (0.05) and *β* (0.80) values, and an effect size of 0.51 as reported in Meade et al., (2018) using MAP as the primary dependent variable in their postexercise hypotension study. Therefore, 14 healthy, normotensive male participants were recruited. Although recruitment was intended to target both males and females, the three females that initially took part in the study dropped out halfway through the training. This study was approved by the Hubei Normal University Ethics committee (No.435) and performed in accordance with the latest version of the Declaration of Helsinki and registration with the China Clinical database (ChiCTR2200065375). Each participant provided verbal and written informed consent. Participants physical characteristics at pre‐training, post‐training and following detraining are summarized in Table 1.

**TABLE 1:** Physical characteristics at Pre, Post and Detraining (Mean (SD), *N* = 14). Data were analyzed by one‐way repeated measures ANOVA.

	Pre	Post	Detraining
Age (years)	25 (3)	25 (3)	25 (3)
Height (cm)	177.2 (6.1)	‐	‐
Weight (kg)	72.9 (9.3)	72.9 (8.8)	73.1 (9.2)
% Body fat	19.5 (4.0)	19.7 (4.1)	20 (3.7)
*V*O_2max_ (mL kg^−1^ min^−1^)	42.8 (6.5)	52.2 (8.0)**	44.5 (5.6)
Peak power output (W)	270.4 (28.8)	322.1 (29.1)**	286.4 (26.4)***

** indicates significant difference between Post‐training and Detraining (*p* < 0.01);

*** indicates significant difference between Pre‐training and Detraining (*p* < 0.05).

### 2.2 Experimental overview

All participants attended the experimental trials that spanned a period of 66 days which included the following visits: one familiarization session, one preliminary testing session, pre‐test assessments (Pre), 20 sessions of high intensity exercise over the course of 4‐weeks, post‐training assessments (Post), a detraining period consisting of 4‐weeks of complete cessation of exercise training, and post‐detraining assessments (Detraining). The pre‐training, post‐training and the post‐detraining trials consisted of a trial with (MP) and without MP (CON) in a counterbalanced order, with each trial separated by 48 h. The postexercise hypotension protocol in the pre‐, post‐training, and post‐detraining assessments consisted of 15 min exercise at a power output associated with 40% of the difference between the gas exchange threshold (GET) and *V̇*O_2max_ (i.e., Δ40%), followed by 1 h recovery in a seated position in a temperate environment (25 ± 0.5°C, 50 ± 6% relative humidity). All trials were performed at the same time of day to minimize the effect of diurnal rhythm on blood pressure and body temperature fluctuations (Millar‐Craig et al., 1978), and were performed 2 h postprandial. Furthermore, all participants were told to abstain from coffee and alcohol intake, as well as avoiding strenuous exercise 48 h prior to each testing.

### 2.3 Preliminary testing

At each time point and following anthropometric measurements, all participants completed a ramp incremental exercise test on a cycle ergometer (Ergoline, USA) for determination of *V̇*O_2max_, the GET, the mean response time (MRT) of oxygen uptake (*V̇*O_2_) kinetics, and subsequently the power output associated with Δ40%. Each test consisted of a 4‐min baseline period of cycling at 20 W, followed by a ramped, linear increase in work rate of 20 W/min at a fixed cadence of 70 rpm until the participant could no longer maintain the required cadence of 60 rpm despite strong verbal encouragement. Ventilatory and gas exchange variables were measured continuously breath‐by‐breath throughout each test. *V̇*O_2max_ was defined as the highest 20 s average obtained at the end of the test, *V̇*O_2max_ was confirmed via the occurrence of a plateau in *V̇*O_2_ with an increasing workload. On a separate day, a validation trial was conducted to ensure the accuracy of the *V̇*O_2max_ measurement, with the difference between both trials being nonsignificant to each other (all *p* > 0.3). The GET and MRT were determined as previously described (Goulding et al., 2017). Δ40% was selected as the exercise intensity for our postexercise hypotension protocol as pilot testing indicated that this intensity was the most effective in eliciting a postexercise hypotension response, while also maintaining the tolerable duration of exercise above 15 min to enable completion of the training sessions.

### 2.4 Familiarization session

Two days following the ramp incremental exercise test, participants attended a familiarization session to ensure that they were familiar with the experimental procedure including exercise at the power output associated with Δ40% elicited the expected physiological responses and could be sustained for 15 min for each participant.

### 2.5 Echocardiography measurement

An echocardiography test was issued 1 day prior to the Pre, Post, and Detraining at the university affiliated hospital between 8 a.m. and 10 a.m. To ensure adequate hydration, all participants consumed 500 mL mineral water prior to the echocardiography test. Echocardiography test was conducted under resting conditions only.

### 2.6 Postexercise hypotension protocol and the skeletal MP

The Pre, Post, and the Detraining assessment protocol consisted of a postexercise hypotension protocol with (MP) and without (CON) maintenance of the MP using the same absolute power output as pre‐training.

One day prior to the postexercise hypotension trial, participants were required to consume a standardized low sodium diet administered by the university cafeteria to prevent diet‐related fluctuations in blood pressure. Participants were required to drink 600 mL water 2 h prior to the postexercise hypotension trials.

Upon arrival at the laboratory, participants first performed arterial stiffness measurements and then self‐inserted a rectal thermistor. Subsequently, participants rested on the cycle ergometer for 40 min during which they were instrumented and resting data were obtained. Following baseline measurements, participants then began 5 min of baseline cycling at 25 W before a step change in power output to the power associated with Δ40% for 15 min, followed by 1 h of recovery at the seated position. Throughout the entire trial, core temperature, skin temperature, and heart rate (HR) were recorded continuously while blood pressure was recorded at the end of exercise and every 5 min during the recovery in duplicate. Expired gas exchange and ventilation were measured continuously during the exercise. Lastly, the participant's rating of perceived exertion (RPE) was measured at the end of the exercise bout. Upon the completion of the recovery, capillary blood samples were obtained and arterial stiffness was measured. The exercise protocol in the skeletal MP trial (MP) was identical to that in CON, with the exception that upon completion of the Δ40% exercise bout, participants performed back pedaling unloaded exercise at 50 revolutions per minute, controlled by a metronome, throughout the 1 h recovery period. We employed back pedaling instead of forward pedaling since forward pedaling had a default 20 W workload while back pedaling had a negligible external workload.

### 2.7 High‐intensity exercise training

One day following the completion of the last postexercise hypotension trial, participants began their high‐intensity exercise training period consisting of five sessions per week on consecutive days at the power output associated with Δ40% for 15 min. To maintain the same relative exercise intensity of Δ40% throughout the training period and to fulfill the principle of progressive overload, a ramp incremental exercise test was conducted at the end of the second week of training and a new Δ40% was calculated and used in the last 2 weeks of the training sessions. The average increase of power output from Week 2 onward was 24 ± 7 W. To standardize the same training volume across every participant, participants were instructed not to perform any additional training outside of the laboratory sessions. Upon the completion of 4 weeks of training, an echocardiography scan and a ramp incremental exercise test were performed prior to the postexercise hypotension protocol. The ramp incremental exercise test and the postexercise hypotension protocol were separated by 48 h.

### 2.8 Detraining

Upon the completion of the last postexercise hypotension trial, participants began their detraining phase. During this phase, participants were required not to perform any structured physical exercise training and only daily commuting activities were allowed during this 4 week period. This control of physical activity was confirmed by the participants via a questionnaire on a daily basis. Upon the completion of detraining, an echocardiography scan and a *V̇*O_2max_ test were performed prior to the postexercise hypotension protocol.

### 2.9 Measurements

#### 2.9.1 Anthropometric

Height and body mass were measured using a stadiometer (Seca, Hamburg, Germany; accurate to 0.1 cm) and scale (Jadever, Taipei, Taiwan; accurate to 10 g), from which body surface area was estimated (25). Percentage body fat was measured via bioelectrical impedance (InBody 230, Seoul, Korea).

#### 2.9.2 Respiratory

Expired respiratory gases were collected from a mixing chamber and analyzed for *V̇*O_2_ and carbon dioxide elimination (*V̇*CO_2_), ventilation (*V̇*E) and respiratory exchange ratio (RER), using an online, breath‐by‐breath system (Max II, AEI, USA). Data were recorded breath‐by‐breath and smoothed using a 30 s average. This system was calibrated before each trial using a zero and *β*‐standard gas concentrations, and a 3‐L syringe (Hans Rudolph 3 L Calibration Syringe).

#### 2.9.3 Cardiovascular

HR was recorded from the detection of R–R intervals (Polar Vantage XL, Polar Electro, Kempele, Finland) while brachial artery blood pressure was measured manually via sphygmomanometry, in duplicate by the same experienced operator. Mean arterial pressure (MAP) was calculated as diastolic blood pressure + 1/3 pulse pressure. Vascular stiffness was measured by brachial ankle pulse wave velocity (baPWV) as a surrogate of arterial compliance. It was previously shown that the greater the gradient between the baseline baPWV and the end of exercise baPWV, the greater the increase of arterial compliance (Kingwell et al., 1997). We therefore used the change of baPWV from baseline as an index of the change of vascular compliance at Pre, Post, and Detraining as well as between MP and CON. Due to machine malfunction, one participant did not complete the baPWV and thus 13 participants were used for this analysis. Resting cardiac function variables such as ejection fraction, fractional shortening velocity, arcus aorta diameter, size of the left ventricle and left atrium were measured by the same sonographer using 2‐D echocardiography over two cardiac cycles using (Affiniti 70, Philips, the Netherlands). Echocardiography was performed in triplicate using M mode at parasternal short and long axis views.

#### 2.9.4 Body temperatures

Core temperature (*T*_core_) was measured by rectal thermistor (TMQ‐DAG, Unimed, China; accurate to 0.1°C). Mean skin temperature (*T*_sk_) was measured at four sites using calibrated skin thermistors (Grant Instrument Ltd, Cambridge, UK; accurate to 0.2°C) secured on the calf, thigh, chest, and forearm using surgical tape (3 M Healthcare, USA). One participant (the same participant in the baPWV) could not insert the rectal thermistor and thus 13 participants were used for the data analysis. Furthermore, all statistical analyses were completed with and without this participant and omitting him had no impact on the magnitude or direction of statistical results. Area‐weighted mean $\bar{T}$_sk_ was calculated according to the equation of Ramanathan, (1964). Core and skin temperatures were recorded continuously using TracerDAQ software (Measurement Computing Corporation, Norton, MA, USA).

#### 2.9.5 Hematological variables

In each session before, immediately after exercise, and following 1 h recovery, capillary blood samples were taken from the fingertip in duplicate. Whole blood was used to measure hemoglobin concentration (Hemo Control, EKF diagnostics, catalogue number: 300‐3012‐0765; Germany). Subsequently, hematocrit was calculated and the plasma volume changes were calculated as according to Dill & Costill, (1974).

#### 2.9.6 Data and statistical analysis

As postexercise hypotension is defined as the reduction of MAP from baseline, the analysis of systolic blood pressure, diastolic, and MAP between Con and MPP and across different training status (Pre, Post, and Detraining) were analyzed and presented as the change from baseline value. For clarity, absolute blood pressure data are also presented.

All statistical analyses were performed with SPSS software for Windows (IBM SPSS Statistics 20, NY, USA) and figures were produced using GraphPad Prism (Prism version 7.00, GraphPad Software). Homogeneity of variance was examined by Levene's test, and the normality of the data was examined by the Kolmogorov–Smirnov Test. Resting physiological and RPE data between MP and CON and different training stages (pre‐training, post‐training, and detraining), as well as the change of baPWV from baseline at the same training stages were analyzed by two‐way repeated measures ANOVA. All other data during exercise and following the recovery were analyzed by three‐way repeated measures ANOVA; in cases where main or interaction effects occurred, post hoc pairwise analyses were performed, using paired samples *t*‐tests with Bonferroni correction. All values are reported as means ± standard deviation (SD) unless stated otherwise. Statistical significant level was set at *p* ≤ 0.05.

## 3 RESULTS

### 3.1 Evidence of postexercise hypotension and effect of MP at pre

**FIGURE 1:**
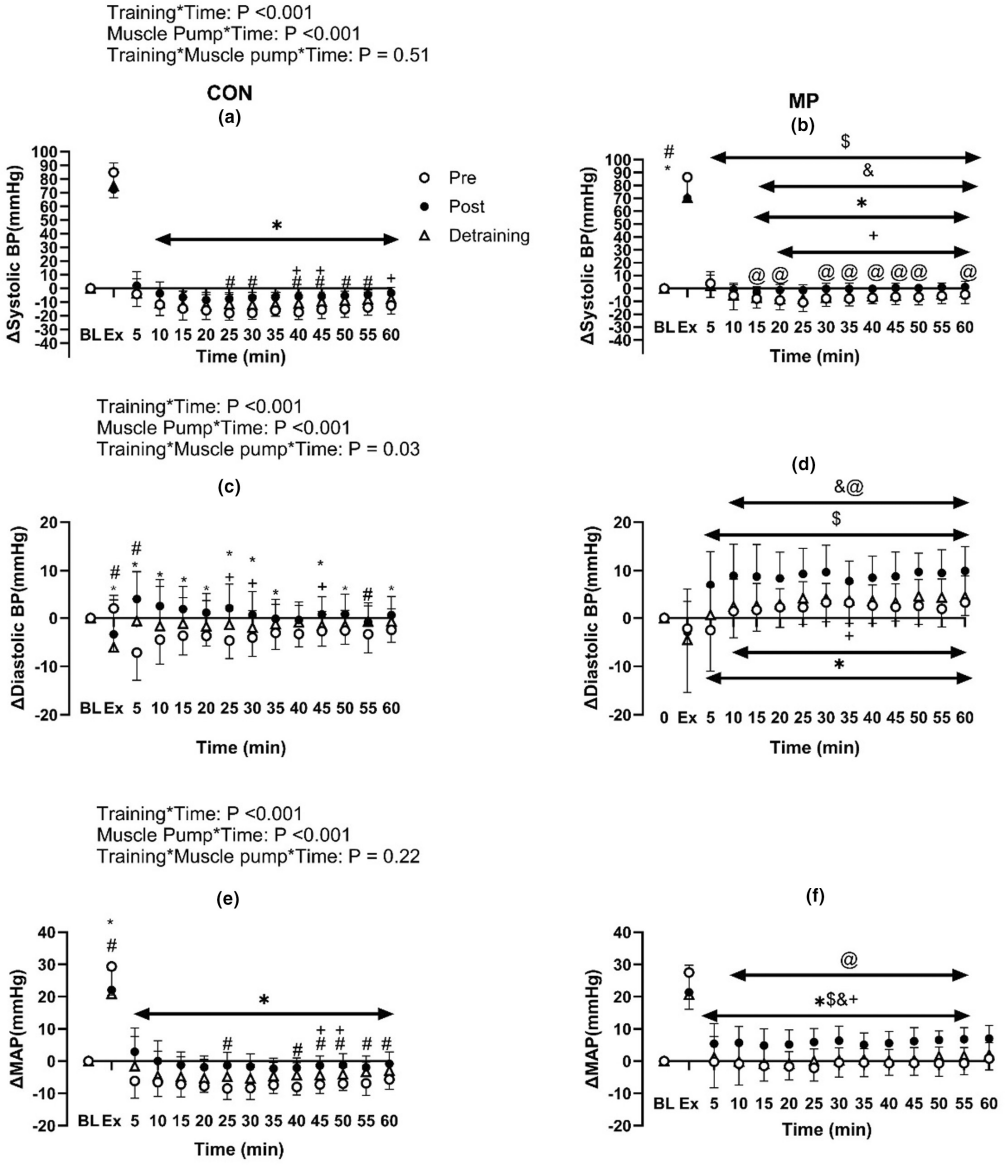
Effect of 4 weeks of high‐intensity exercise training (Post) and detraining with (b, d, f) and without MP (a, c, e on the change of systolic blood pressure (a, b), diastolic blood pressure (c, d) and MAP (e, f) from baseline during exercise and following 1 h of recovery (*N* = 14). * Indicates significant difference between Pre and Post; +indicates significant differences between Post and Detraining; # Indicates significant difference between Pre and Detraining; $Indicates significant difference between corresponding Pre (CON) value (Pre: CON vs. MP); & indicates significant difference between corresponding Post (CON) value (Post: CON vs. MP); @indicates significant differences between corresponding Detraining (CON) value. Baseline data (BL) were analyzed by one‐way repeated ANOVA while data during and following exercise with and without MP across Pre, Post and Detraining were analyzed by three‐way repeated ANOVA.

**FIGURE 2:**
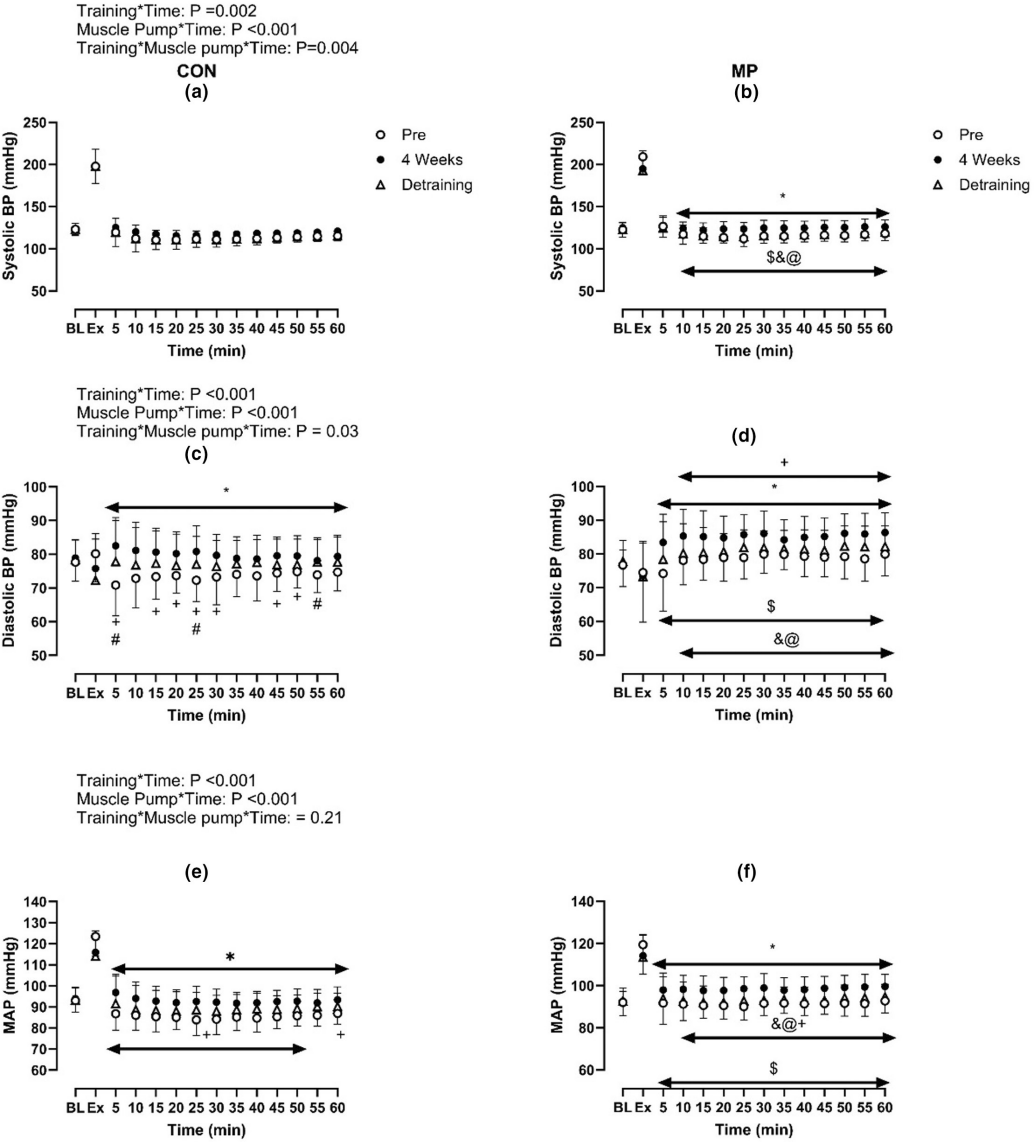
Effect of 4 weeks of high‐intensity exercise training (Post) and detraining with (b, d, f) and without MP (a, c, e on absolute systolic blood pressure (a, b), diastolic blood pressure (c, d), and MAP (e, f) during exercise and following 1 h of recovery (*N* = 14). * Indicates significant difference between Pre and Post; +indicates significant differences between Post and Detraining; # indicates significant difference between Pre and Detraining; $indicates significant difference between corresponding Pre (CON) value (Pre: CON vs. MP); & indicates significant difference between corresponding Post (CON) value (Post: CON vs. MP); @ indicates significant differences between corresponding Detraining (CON) value. Baseline data (BL) were analyzed by one‐way repeated ANOVA while data during and following exercise with and without MP across Pre, Post and Detraining were analyzed by three‐way repeated ANOVA.

Systolic blood pressure, diastolic blood pressure, MAP, HR, and baPWV during exercise were not different between CON and MP (all *p* > 0.20, Figures 1 and 2). Following 1 h recovery in CON, postexercise hypotension was evident as systolic blood pressure, diastolic blood pressure, and MAP were lower than observed at baseline (All *p* < 0.01, Figure 2). MP resulted in a higher systolic blood pressure, diastolic blood pressure, and MAP response from baseline when compared to CON (*p* < 0.01, Figures 1 and 2). Furthermore, HR was elevated in MP compared to CON during recovery between 15 and 45 min (*p* < 0.01). The postexercise changes in baPWV did not differ between MP and CON (*p* = 0.14, *n* = 13).

Hematocrit did not differ between CON and MP at rest, end‐exercise or following recovery (All *p* > 0.1). Hematocrit was elevated following exercise (*p* < 0.01) and remained so throughout recovery (*p* < 0.01). Following recovery, plasma volume was elevated compared to rest (*p* < 0.01) but did not differ between CON and MP (*p* = 0.73). *T*_sk_ during the recovery period was not different between CON and MP (*p* = 0.844). Similarly, *T*_core_ during exercise was not different between CON and MP (*p* = 0.90).

### 3.2 Effect of exercise training

**FIGURE 3:**
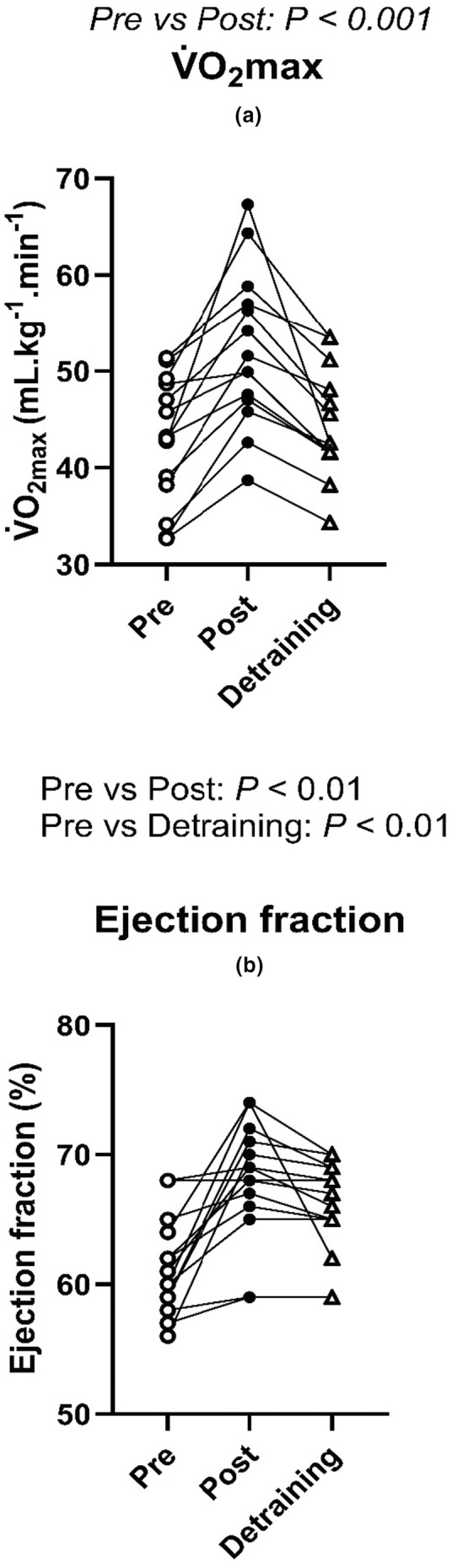
Individual changes in *V̇*O_2max_ (a) and resting ejection fraction (b) following 4 weeks of high intensity training and detraining (*N* = 14). Data were analyzed by one‐way repeated ANOVA.

Following 4 weeks of training, all participants increased their *V̇*O_2max_, resting ejection fraction and fractional shortening velocity (all *p* < 0.01, Tables 1 and 2, Figure 3).

**TABLE 2:** Echocardiography data at Pre‐training, Post‐training and Detraining (Mean (SD), N = 14). Data were analyzed by one‐way repeated measures ANOVA.

	Pre‐training	Post‐training	Detraining
Left atrium (cm)	3.10 (0.25)	3.21 (0.19)	3.05 (0.31)
Left ventricle (cm)	4.63 (0.23)	4.70 (0.50)	4.60 (0.30)
Arcus aorta diameter (cm)	2.76 (0.24)	2.81 (0.24)	2.70 (0.23)
Ejection fraction (%)	61 (0.04)	67.9 (0.05)*	65.9 (0.04)***
Fractional shortening velocity (%)	32.3 (0.03)	38.2 (0.04)**	36 (0.03)***

* Indicates significant difference between Pre‐training and Post‐training (*p* < 0.01);

** indicates significant difference between Post‐training and Detraining (*p* < 0.05);

*** indicates significant difference between Detraining and Pre‐training (*p* < 0.05).

Resting systolic blood pressure, diastolic blood pressure and MAP were not different between Pre and Post training (all *p* > 0.50, Figure 2). Similarly, resting baPWV was not different across Pre and Post (*p* = 0.43). In MP at rest, systolic blood pressure, MAP, HR and baPWV were identical to CON (all *p* > 0.16) while diastolic blood pressure was lower in MP than CON (*p* < 0.05).

**FIGURE 4:**
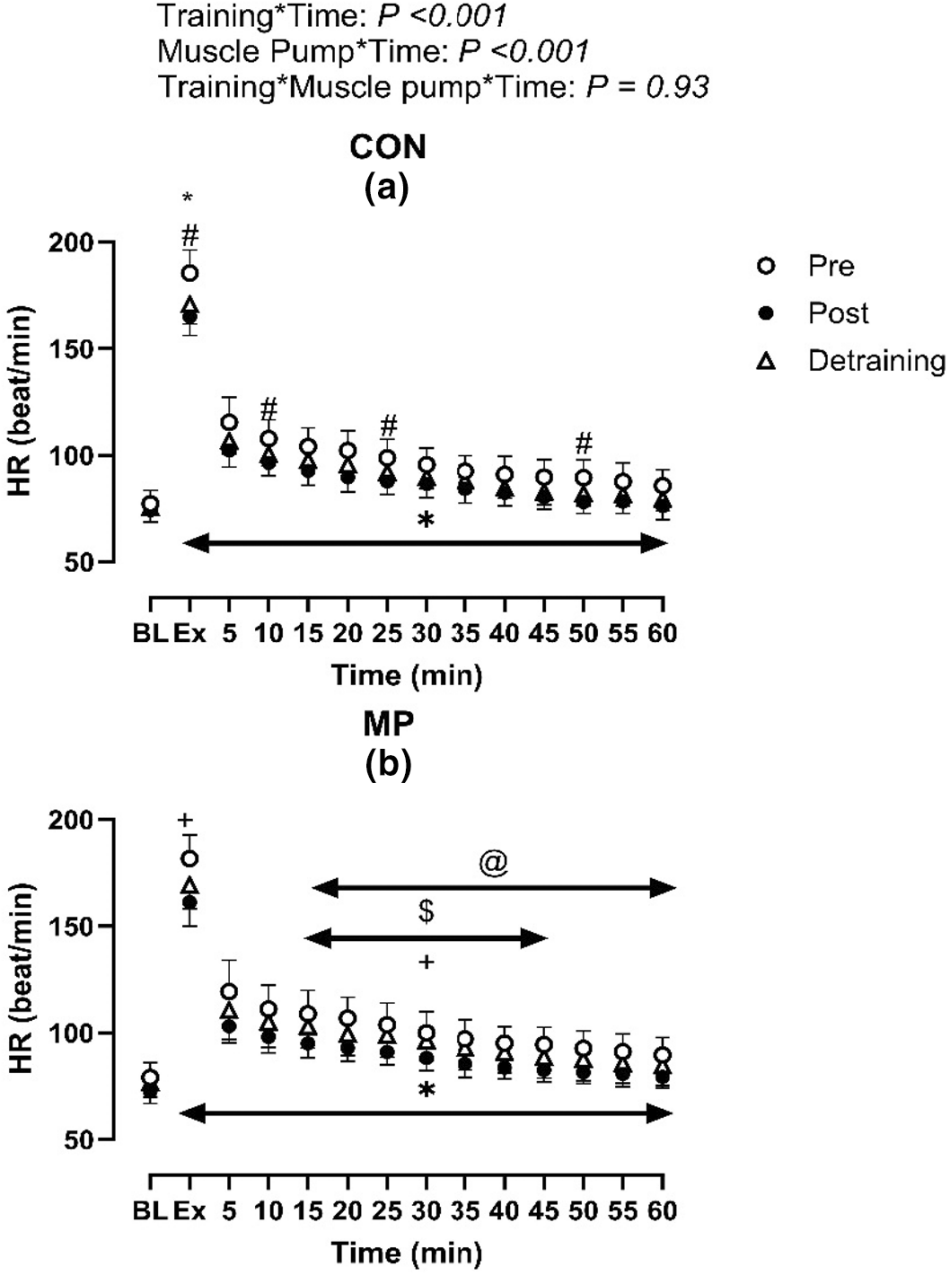
Effect of 4 weeks high‐intensity exercise training (Post) with (b) and without MP (a) on heart rate responses during exercise and following 1 h of recovery (*N* = 14). * Indicates significant difference between Pre and Post; +indicates significant differences between Post and Detraining; # indicates significant difference between Pre and Detraining; $indicates significant difference between corresponding Pre (CON) value (Pre: CON vs. MP); & indicates significant difference between corresponding Post (CON) value (Post: CON vs. MP); @indicates significant differences between corresponding detraining (CON) value. Baseline data (BL) were analyzed by one‐way repeated ANOVA while data during and following exercise with and without MP across Pre, Post and Detraining were analyzed by three‐way repeated ANOVA.

**FIGURE 5:**
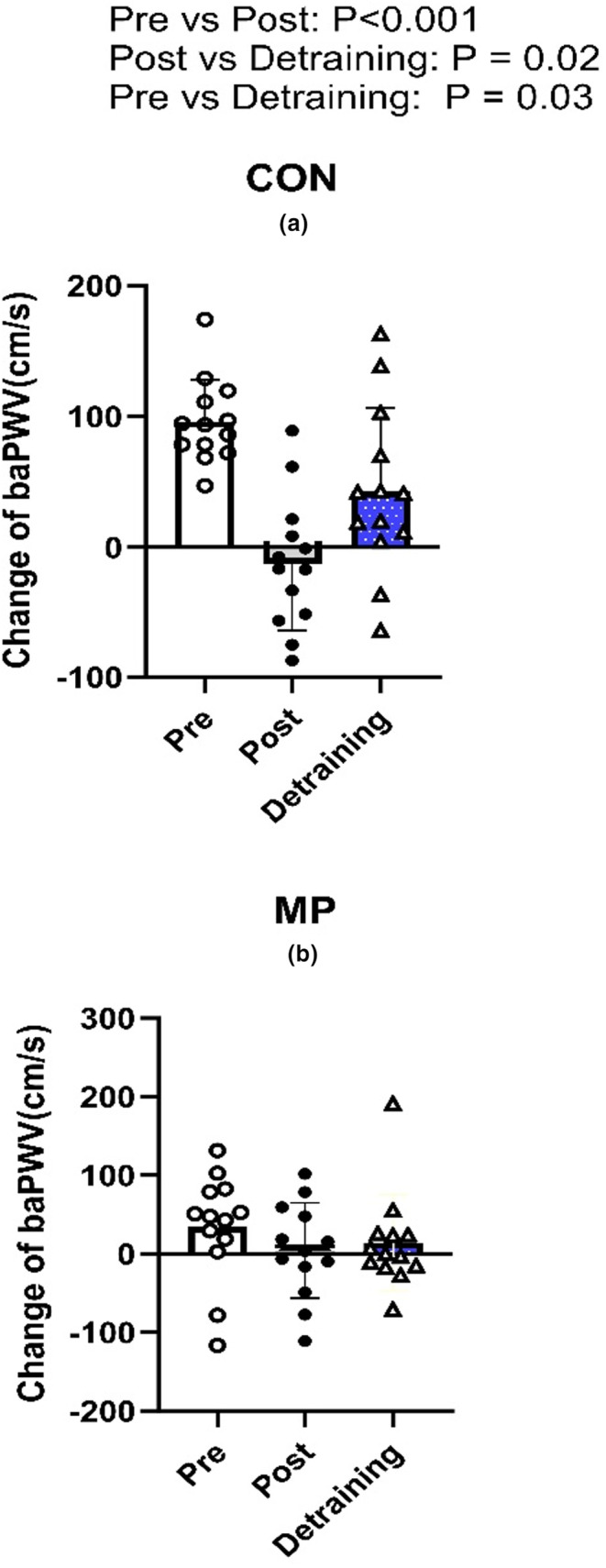
The individual and mean changes of baPWV from baseline at Pre, Post and Detraining with (b) and without MP (a) (*N* = 13). Data were analyzed by one‐way repeated ANOVA.

Following 4 weeks of training, systolic blood pressure, diastolic blood pressure and MAP were elevated during recovery compared to PRE (all *p* < 0.01, Figures 1 and 2). Specifically, the postexercise reductions in systolic blood pressure, diastolic blood pressure, and MAP were smaller than Pre at the majority of time points (Figure 1.). In MP during the 1 h recovery, systolic blood pressure, diastolic blood pressure and MAP did not differ from baseline during recovery, demonstrating that MP abolished postexercise hypotension post‐training (all *p* > 0.05). Moreover, these variables were elevated compared to both CON and Pre (all *p* < 0.05). HR during the recovery was lower than Pre in CON and in MP throughout the recovery period (all *p* < 0.01, Figure 4). Lastly, the change of baPWV from baseline was larger in Pre than Post in CON only (*p* < 0.01, *n* = 13, Figure 5).

**FIGURE 6:**
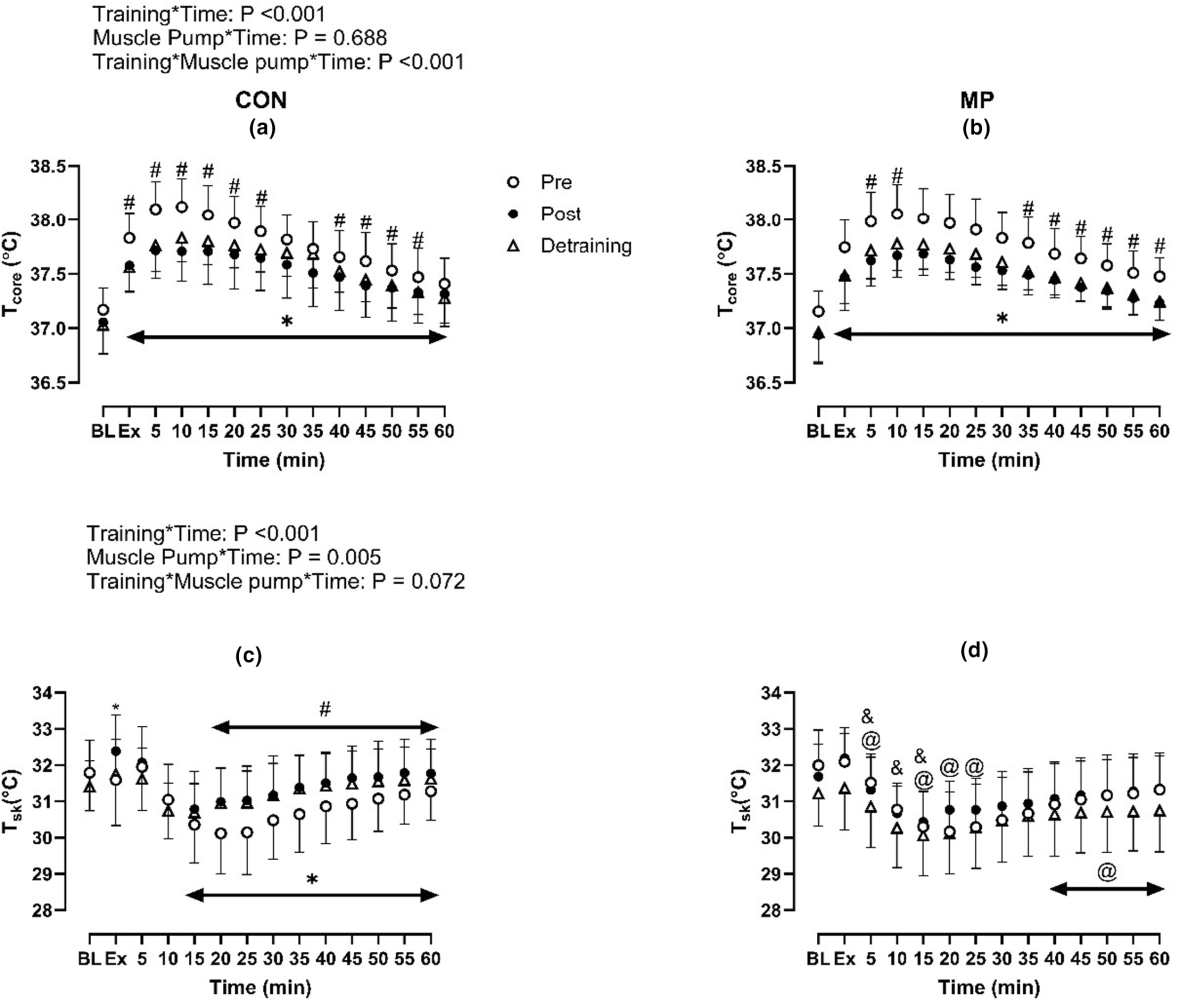
Effect of 4 weeks high‐intensity exercise training (Post) and detraining with (b, d) and without MP (a, c) on core and skin temperature responses during exercise and during 1 h of the recovery (*N* = 13). * Indicates significant difference between Pre and Post; #indicates significant difference between Pre and Detraining; & indicates significant difference between corresponding Post (CON) value (Post: CON vs. MP); @indicates significant differences between corresponding Detraining (CON) value (Detraining: CON vs. MP). Baseline data (BL) were analyzed by one‐way repeated ANOVA while data during and following exercise with and without MP across Pre, Post and Detraining were analyzed by three‐way repeated ANOVA.

Following training, the T_sk_ response was greater than Pre in CON at the majority of time points during recovery (*p* < 0.01, Figure 6). Conversely, T_core_ was reduced during recovery in both CON and MP when compared to Pre (*p* < 0.01). T_core_ was not different between CON and MP trials (*p* = 0.34).

Both resting and postexercise hematocrit did not differ between Pre and Post or between CON and MP (all *p* > 0.40). Similarly, plasma volume changes did not differ between Pre and Post or between CON and MP (all *p* > 0.5).

### 3.3 The effect of detraining

Detraining resulted in a lower *V̇*O_2max_, ejection fraction and fractional shortening velocity when compared to Post (all *p* < 0.01, Figure 3). Although *V̇*O_2max_ returned to similar levels to Pre (*p* > 0.05), ejection fraction and fractional shortening velocity remained higher than Pre (all *p* < 0.05, Table 2, Figure 3).

Resting absolute systolic blood pressure, diastolic blood pressure and MAP were not different across Pre, Post, and Detraining (all *p* > 0.50, Figure 2). Similarly, resting baPWV was not different across Pre, Post, and Detraining (*p* = 0.43). In the MP condition at rest, the systolic blood pressure, diastolic blood pressure, MAP, HR, and baPWV were identical to the CON (all *p* > 0.30).

The reductions in systolic blood pressure and MAP from rest in CON were greater following Detraining when compared to Post (all *p* < 0.05, Figures 1 and 2). When compared to Pre, the postexercise changes in systolic blood pressure, diastolic blood pressure and MAP were less following Detraining in CON (all *p* < 0.05). Furthermore, the changes of systolic blood pressure, diastolic blood pressure, and MAP from rest in MP were reduced following Detraining when compared to Post (all *p* < 0.05). The increases in systolic blood pressure, diastolic blood pressure, and MAP from rest were higher in the MP than the corresponding Detraining time points in CON.

Heart rate during the recovery in CON was lower in Detraining than Pre (*p* < 0.05, Figure 4) but was not different between Detraining and Post (*p* > 0.05). Furthermore, HR was elevated in MP versus CON within Detraining timepoints. Lastly, the change of baPWV in CON from baseline was larger in Detraining than Post (*p* < 0.05, Figure 5) but was smaller compared to Pre.

*T*_sk_ did not differ between Detraining and any other time point (All *p* > 0.1), whereas MP resulted in a lower *T*_sk_ response compared to CON following Detraining (*p* < 0.01, Figure 6). *T*_core_ in CON and MP were lower than most of the timepoints when compared to Pre‐during the recovery (*p* = 0.02, Figure 5) but were not different between CON and MP (*p* = 0.34).

Baseline hematocrit was higher in Detraining versus Post in both CON and MP trials (All *p* < 0.05), however, end‐exercise hematocrit was greater in Detraining than Post in CON only (*p* < 0.05). Postexercise changes in plasma volume did not differ between Detraining and any other time point for both CON and MP (all *p* > 0.05).

## 4 DISCUSSION

The principal original findings of this investigation were as follows: (1) 4‐week of high‐intensity exercise training reduced the magnitude of postexercise hypotension; (2) the effect of training on postexercise hypotension was partially retained for at least 4 weeks following the complete cessation of any physical training; and (3) training performed alongside MP completely nullified the occurrence of postexercise hypotension. These findings agree with our study hypotheses. Collectively, this study indicates that training status greatly alters the magnitude of postexercise hypotension in a healthy male population.

We observed that 4‐week of high‐intensity exercise training was able to reduce the magnitude of postexercise hypotension response (Figure 1). This observation is similar to Rivas et al., (2019) where it was shown that 10‐days of physical training in a thermoneutral environment was able to attenuate the magnitude of postexercise hypotension in the final 30 min of the recovery. We found that the baPWV changes from rest were reduced following training (Figure 5), and resting ejection fraction was increased (Figure 3; Table 2). Hence, a plausible mechanism for the reduction in postexercise hypotension following training might be a combination of reduced postexercise arterial compliance and an increase in resting ejection fraction and fractional shortening velocity following training. Assuming this elevated ejection fraction at rest was maintained during recovery from exercise, this would enable a greater MAP during recovery. However, this increase of ejection fraction must be due to an increase in cardiac contractility and not from the expansion of plasma volume, as we observed no changes in hematocrit or plasma volume following 4‐weeks of exercise training and detraining.

The study by Rivas et al., (2019) previously demonstrated that 10 days of exercise training in a temperate environment resulted in a lower magnitude of postexercise hypotension during recovery. However, as the exercise intervention employed therein did not measure *V̇*O_2max_, the independent effects of training status per se on the magnitude of postexercise hypotension could not be determined. Moreover, the results of the present study highlight a plausible physiological mechanism pertaining to the reduction of postexercise hypotension following high‐intensity exercise training (i.e., improved cardiac function and reduced postexercise vasodilation which enabled the maintenance of MAP throughout recovery). Furthermore, this is the first original study to examine the effect of detraining on postexercise hypotension. Specifically, our findings reveal that the attenuation of the postexercise hypotension response can be retained for at least 4 weeks following complete cessation of any physical training. As ejection fraction and fractional shortening velocity at rest (Figure 3; Table 2) remained elevated and baPWV changes remained reduced following Detraining compared to Pre (Figure 5), the retention of the training effect on postexercise hypotension following detraining is again likely explained by a combination of reduced postexercise vasodilation and improved cardiac function enabling maintenance of greater MAP during recovery.

This is the first study to demonstrate that 4‐weeks of high‐intensity exercise training can greatly attenuate the effect of postexercise hyperthermia (Figure 6). The reduction of *T*_core_ during the recovery period might be expected to result in a lower level of postexercise hyperemia as the magnitude of hyperemia is related to the increase of *T*_core_ (Pearson et al., 2011). This could thereby reduce the magnitude of acute and sustained postexercise vasodilation (Pearson et al., 2011). Our results are in support of this proposed physiological mechanism as we showed that the change of baPWV from resting was reduced when compared to Pre. This effect was retained following Detraining, as both baPWV and *T*_core_ changes remained lower than Pre following Detraining (Figures 5 and 6). However, it is worth acknowledging that we did not directly quantify this with thermodilution technique. This mechanism therefore warrants further investigation. Moreover, our results also show that MP reduces the magnitude of postexercise hypotension despite no differences in *T*_core_ between MP and Con. Hence, while this observation does not rule out a role for *T*_core_ in determining the postexercise hypotension effect, it does demonstrate that the regulation of this physiological phenomenon is multifactorial and could suggest that redundant control mechanisms are at play.

We have also observed that, when combined with MP, exercise training completely nullified the occurrence of postexercise hypotension. This is likely explained by the fact that maintenance of MP during recovery enables greater venous return during recovery due to vascular compression within the skeletal musculature (Carter 3rd et al., 1999). This increase of venous return by MP combined with greater cardiac contractility following high‐intensity exercise training ultimately results in the preservation of MAP throughout the ensuing recovery period.

### 4.1 Limitations

While this study is the first study to reveal the effect of physical training and detraining on postexercise hypotension, this study has two major limitations which warrants further investigation. We did not obtain cardiac output throughout the recovery period and thus could not quantify the dynamic changes of cardiac output at different training statuses. However, as we observed that the recovery MAP was higher in Post and Detraining when compared to Pre, and also given the fact that resting ejection fraction was higher in Post and Detraining when compared to Pre, it is therefore likely that stroke volume was elevated Post and Detraining during the recovery period to maintain the elevated MAP and cardiac output in order to mitigate the reduced HR response during the recovery period. Second, this study used only male participants, hence whether these findings can be applied to females remains unknown and thus warrants further investigation. Future studies should address whether sex influences the effects of exercise training and detraining on postexercise hypotension.

### 4.2 Implications

This study demonstrates that high‐intensity exercise training protects cardiac function even following 4‐week of detraining and thus even short periods of exercise training may confer cardioprotective effects following training cessation.

## 5 CONCLUSION

In conclusion, high‐intensity exercise training combined with skeletal MP can abolish the occurrence of postexercise hypotension, and this effect is partially retained for 4 weeks following the complete cessation of any exercise in healthy young men.

## AUTHOR CONTRIBUTIONS

Tze‐Huan Lei, Richie P Goulding and Narihiko Kondo, Naoto Fujii were responsible for the conception and the experimental design of this study. I‐Lin Wang, Yi‐Ming Chen, Xiao Zhang, and Tze‐Huan Lei were responsible for data collection, data analysis and interpretation of this study. Tze‐Huan Lei and RPG drafted the manuscript. All authors were responsible for interpretation, editing and revising the manuscript. All authors approved the final version of this manuscript. There are no conflicts of interest to report.

## Ethics Statement

Subjects gave written informed consent to the current protocol that was approved by the Hubei Normal University Institutional Review Board and conducted in accordance with the Declaration of Helsinki.

## Data Availability

Data will be available upon requested by the readers.

## References

[phy215862-bib-0001] Bonsu, B. , & Terblanche, E. (2016). The training and detraining effect of high‐intensity interval training on post‐exercise hypotension in young overweight/obese women. European Journal of Applied Physiology, 116, 77–84. 26293124 10.1007/s00421-015-3224-7

[phy215862-bib-0002] Carter, R., 3rd , Watenpaugh, D. E. , Wasmund, W. L. , Wasmund, S. L. , & Smith, M. L. (1999). Muscle pump and central command during recovery from exercise in humans. Journal of Applied Physiology (1985), 87(4), 1463–1469. 10.1152/jappl.1999.87.4.1463 10517779

[phy215862-bib-0003] Chen, C. Y. , & Bonham, A. C. (2010). Postexercise hypotension: Central mechanisms. Exercise and Sport Sciences Reviews, 38, 122–127. 20577060 10.1097/JES.0b013e3181e372b5 PMC2936915

[phy215862-bib-0004] Dill, D. B. , & Costill, D. L. (1974). Calculation of percentage changes in volumes of blood, plasma, and red cells in dehydration. Journal of Applied Physiology, 37, 247–248. 4850854 10.1152/jappl.1974.37.2.247

[phy215862-bib-0005] Elsner, R. W. , & Carlson, L. D. (1962). Postexercise hyperemia in trained and untrained subjects. Journal of Applied Physiology, 17, 436–440.

[phy215862-bib-0006] Gagnon, D. , Jay, O. , Reardon, F. D. , Journeay, W. S. , & Kenny, G. P. (2008). Hyperthermia modifies the nonthermal contribution to postexercise heat loss responses. Medicine and Science in Sports and Exercise, 40, 513–522. 18379215 10.1249/MSS.0b013e31815eb7b8

[phy215862-bib-0007] Goulding, R. P. , Roche, D. M. , & Marwood, S. (2017). Prior exercise speeds pulmonary oxygen uptake kinetics and increases critical power during supine but not upright cycling. Experimental Physiology, 102, 1158–1176. 28627041 10.1113/EP086304

[phy215862-bib-0008] Graham, M. J. , Lucas, S. J. , Francois, M. E. , Stavrianeas, S. , Parr, E. B. , Thomas, K. N. , & Cotter, J. D. (2016). Low‐volume intense exercise elicits post‐exercise hypotension and subsequent hypervolemia, irrespective of which limbs are exercised. Frontiers in Physiology, 7, 199. 27303310 10.3389/fphys.2016.00199 PMC4885852

[phy215862-bib-0009] Grassi, G. , Seravalle, G. , Colombo, M. , Bolla, G. , Cattaneo, B. M. , Cavagnini, F. , & Mancia, G. (1998). Body weight reduction, sympathetic nerve traffic, and arterial baroreflex in obese normotensive humans. Circulation, 97, 2037–2042. 9610534 10.1161/01.cir.97.20.2037

[phy215862-bib-0010] Halliwill, J. R. , Sieck, D. C. , Romero, S. A. , Buck, T. M. , & Ely, M. R. (2014). Blood pressure regulation X: What happens when the muscle pump is lost? Post‐exercise hypotension and syncope. European Journal of Applied Physiology, 114, 561–578. 24197081 10.1007/s00421-013-2761-1 PMC3944103

[phy215862-bib-0011] Kapilevich, L. V. , Kologrivova, V. V. , Zakharova, A. N. , & Mourot, L. (2020). Post‐exercise endothelium‐dependent vasodilation is dependent on training status. Frontiers in Physiology, 11. 10.3389/fphys.2020.00348 PMC7227416 32457640

[phy215862-bib-0012] Kingwell, B. A. , Berry, K. L. , Cameron, J. D. , Jennings, G. L. , & Dart, A. M. (1997). Arterial compliance increases after moderate‐intensity cycling. The American Journal of Physiology, 273, H2186–H2191. 9374752 10.1152/ajpheart.1997.273.5.H2186

[phy215862-bib-0013] MacDonald, J. R. , MacDougall, J. D. , & Hogben, C. D. (1999). The effects of exercise intensity on post exercise hypotension. Journal of Human Hypertension, 13, 527–531. 10455474 10.1038/sj.jhh.1000866

[phy215862-bib-0014] Maggioni, M. A. , Castiglioni, P. , Merati, G. , Brauns, K. , Gunga, H. C. , Mendt, S. , Opatz, O. S. , Rundfeldt, L. C. , Steinach, M. , Werner, A. , & Stahn, A. C. (2018). High‐intensity exercise mitigates cardiovascular deconditioning during long‐duration bed rest. Frontiers in Physiology, 9, 1553. 30510516 10.3389/fphys.2018.01553 PMC6252355

[phy215862-bib-0015] Meade, R. D. , Crandall, C. G. , Gagnon, D. , & Kenny, G. P. (2018). Greater fluid loss does not fully explain the divergent hemodynamic balance mediating postexercise hypotension in endurance‐trained men. Journal of Applied Physiology, 1985(124), 1264–1273. 10.1152/japplphysiol.00988.2017 PMC6008076 29389247

[phy215862-bib-0016] Millar‐Craig, M. , Bishop, C. , & Raftery, E. B. (1978). Circadian variation of blood pressure. The Lancet, 311, 795–797. 10.1016/s0140-6736(78)92998-7 85815

[phy215862-bib-0017] Mündel, T. , Perry, B. G. , Ainslie, P. N. , Thomas, K. N. , Sikken, E. L. G. , Cotter, J. D. , & Lucas, S. J. E. (2015). Postexercise orthostatic intolerance: Influence of exercise intensity. Experimental Physiology, 100, 915–925. 26040636 10.1113/EP085143

[phy215862-bib-0018] Pearson, J. , Low, D. A. , Stöhr, E. , Kalsi, K. , Ali, L. , Barker, H. , & González‐Alonso, J. (2011). Hemodynamic responses to heat stress in the resting and exercising human leg: Insight into the effect of temperature on skeletal muscle blood flow. American Journal of Physiology. Regulatory, Integrative and Comparative Physiology, 300, R663–R673. 21178127 10.1152/ajpregu.00662.2010 PMC3064274

[phy215862-bib-0019] Pescatello, L. S. , Fargo, A. E. , Leach, C. N., Jr. , & Scherzer, H. H. (1991). Short‐term effect of dynamic exercise on arterial blood pressure. Circulation, 83, 1557–1561. 2022015 10.1161/01.cir.83.5.1557

[phy215862-bib-0020] Ramanathan, N. L. (1964). A new weighting system for mean surface temperature of the human body. Journal of Applied Physiology, 19, 531–533. 14173555 10.1152/jappl.1964.19.3.531

[phy215862-bib-0021] Rivas, E. , Crandall, C. G. , Suman, O. E. , Moustaid‐Moussa, N. , & Ben‐Ezra, V. (2019). Exercise heat acclimation causes post‐exercise hypotension and favorable improvements in lipid and immune profiles: A crossover randomized controlled trial. Journal of Thermal Biology, 84, 266–273. 31466764 10.1016/j.jtherbio.2019.07.017

[phy215862-bib-0022] Winker, R. , Barth, A. , Bidmon, D. , Ponocny, I. , Weber, M. , Mayr, O. , Robertson, D. , Diedrich, A. , Maier, R. , Pilger, A. , Haber, P. , & Rüdiger, H. W. (2005). Endurance exercise training in orthostatic intolerance: A randomized, controlled trial. Hypertension, 45, 391–398. 15699447 10.1161/01.HYP.0000156540.25707.af

